# Recent advances in cultivation-independent molecular-based techniques for the characterization of vaginal eubiosis and dysbiosis

**DOI:** 10.12703/r/9-21

**Published:** 2020-12-09

**Authors:** Ronald F Lamont, Ellen HA van den Munckhof, Birgitte Møller Luef, Christina Anne Vinter, Jan Stener Jørgensen

**Affiliations:** 1 Department of Gynecology and Obstetrics, University of Southern Denmark, Institute of Clinical Research, Research Unit of Gynaecology and Obstetrics, Kløvervænget 10, 10th floor, 5000 Odense C, Denmark; 2 Division of Surgery, University College London, Northwick Park Institute of Medical Research Campus, London, HA1 3UJ, UK; 3 DDL Diagnostic Laboratory, Visseringlaan 25, 2288 ER Rijswijk, The Netherlands

**Keywords:** Bacterial Vaginosis, Diagnosis, Molecular Test, Real-Time PCR, Sensitivity, Specificity, Vaginal Dysbiosis, Vaginal Eubiosis

## Abstract

“The bacterial vaginosis syndrome” has significant adverse effects for women and babies, including preterm birth and increased risk of acquisition of sexually transmitted infections and HIV. Currently, the gold standard for diagnosis is Gram stain microscopy of vaginal secretions, which is not readily available, is somewhat subjective, and does not differentiate between the likely different subtypes of vaginal dysbioses that may have different etiologies, microbiology, responses to antibiotics, and phenotypic outcomes. With new information from molecular-based, cultivation-independent studies, there is increasing interest in the use of molecular techniques for the diagnosis of bacterial vaginosis. We reviewed the current evidence on and the rationale behind the use of molecular techniques for the diagnosis of bacterial vaginosis. We found a number of commercially available molecular diagnostic tests, a few of which have US Food and Drug Administration (FDA) and/or Conformité Européenne *in vitro* diagnostic (CE-IVD) approval, and we have compared their performance with respect to sensitivities and specificities. Molecular-based tests have the advantage of objectivity, quantification, detection of fastidious organisms, and validity for self-obtained vaginal swabs. The performance of the molecular tests against standard microscopy is impressive, but further education of users on interpretation is needed. Bacterial vaginosis is the major cause of vaginal dysbiosis and should be recognized for the threat it is to women’s genital tract health. Quantitative assessment of microbial abundance, the diversity of other organisms present, specific primers for gene sequence regions, and clades and biovars of target microbes should be recognized and incorporated into future molecular diagnostic tests to better differentiate between vaginal eubiosis and dysbiosis.

## Background

### Vaginal eubiosis and dysbiosis

For the purpose of this review, we have defined vaginal eubiosis as the presence in the vagina of a beneficial lactic-acid-producing microbiota, predominantly, but not uniquely, from the genus *Lactobacilli.* These organisms provide vaginal eubiosis *inter alia* through i) numerical dominance, ii) vaginal epithelial cell adhesion that prevents biofilm formation, and iii) killing ability over dysbiotic organisms through the production of lactic acid, hydrogen peroxide (H_2_O_2_), and naturally occurring antimicrobials such as bacteriocins^1,2^.

Vaginal dysbiosis may be defined as a prolonged deviation from a low-diversity, *Lactobacillus*-abundant/-dominant vaginal microbiota to a microbiota that has a high diversity and high abundance of potentially pathogenic organisms. This deviation may be due to the introduction of abnormal bacteria into the vagina and a paucity of *Lactobacilli*, particularly *Lactobacillus crispatus, Lactobacillus jensenii*, and *Lactobacillus gasseri.* This dysbiosis may be by sexual transmission of organisms such as *Chlamydia trachomatis* or *Neisseria gonorrhoeae* or by non-sexual transmission with organisms such as group A or G *Streptococcus.* Another possibility is that a pre-existing commensal such as *Gardnerella vaginalis* or *Escherichia coli* increases their virulence^3,4^. Finally, there may an imbalance of the normal vaginal microbiota whereby *Lactobacilli* are reduced in quality and/or quantity, the vaginal pH rises, and there is a 1,000-fold increase in the number of other potentially pathogenic organisms such as *G. vaginalis, Atopobium vaginae, Megasphaera, Sneathia, Candidatus Lachnocurva vaginae* (formerly known as BV-associated bacterium [BVAB]-1)^5^, *Mageeibacillus indolicus* (formerly known as BVAB-3)^6^, *Mobiluncus*, and other anaerobes. This latter scenario is known as bacterial vaginosis (BV) and is the commonest cause of vaginal discharge in high-income countries, responsible for one-third of all vaginal infectious morbidity worldwide, and a major cause of adverse sequelae in obstetrics and gynecology^7^. Historically, BV has been poorly understood because the etiology remains unknown, the microbiology differs between subjects, the response to antibiotics differs, recurrence and resistance to treatment are common, and phenotypic outcomes of the condition vary. While historically BV has been considered by some to be a mild inconvenience for women, we now know that through human immunodeficiency virus (HIV), preterm birth (PTB), and sexually transmitted infections (STIs) it carries a significant mortality and morbidity for women and babies.

In a cross-sectional study of 394 healthy women of reproductive age, subsequently elaborated by others, the human vaginal microbial communities were classified into five community state types (CSTs): i) CST-I dominated by *L. crispatus*, ii) CST-II, dominated by *L. gasseri*, iii) CST-III dominated by *Lactobacillus iners*, iv) CST-V dominated by *L. jensenii*, and v) CST-IV, which has no specific dominant species and was termed the diverse group, characterized by a paucity of *Lactobacilli* and higher proportions of strictly anaerobic bacteria, including *Prevotella, Dialister, Atopobium, Gardnerella, Megasphaera, Peptoniphilus, Sneathia, Eggerthella, Aerococcus, Finegoldia,* and *Mobiluncus*. CST-IV is further subdivided into CST-IVa and CST-IVb, both of which lack *Lactobacilli* and contain an abundance of such anaerobes or other BV-associated organisms^8^. Many feel that there are numerous other dysbiotic CSTs like Ravel’s CST-IV, which may explain the poor understanding of BV with respect to different etiologies, microbiologies, responses to antibiotics, recurrence, and phenotypic outcomes^7,9^.

BV is an important condition associated with a number of adverse sequelae in both obstetrics and gynecology. In pregnancy, BV has been associated with early, late, and recurrent miscarriage, post-abortal sepsis, preterm prelabor rupture of the membranes (PPROM), postpartum endometritis, chorioamnionitis, and PTB^7^. In gynecology, BV has been associated with urinary tract infection, pelvic inflammatory disease, post-hysterectomy vaginal cuff infection, infertility, and acquisition of bacterial, protozoal, and viral STIs, such as herpes simplex virus (HSV), human papilloma virus (HPV), and HIV. In 1955, Gardner and Dukes’ Haemophilus vaginitis (renamed BV in 1984) was considered to be nothing more than a mild inconvenience for women^10^, and in our experience this continues to be the case. Since 50% of women with BV are asymptomatic, this myth is perpetuated by government agencies (see https://www.nhs.uk/conditions/bacterial-vaginosis/).

In 2012, because of concerns over increasing antibiotic resistance, and as an incentive for pharmaceutical companies to develop new antibiotics, the Generating Antibiotic Incentives Now (GAIN) Act was introduced into US law as a part of the US FDA Safety and Innovation Act. This legislation extends by 5 years, from 15 to 20, the time in which antibiotics for serious or life-threatening infections can be sold without generic competition. The GAIN Act grants fast-track and priority review status as well as an expedited regulatory approval process to qualifying drugs^11^. Secnidazole, a new antibiotic used to treat BV, was initially rejected by the FDA for processing through the GAIN Act; however, over the last 2 years, BV has been recognized as a serious or life-threatening disease that meets the criteria for fast tracking through the GAIN Act, and secnidazole is now an approved treatment for BV in the US (see https://www.catalent.com/index.php/news-events/news/Symbiomix-Therapeutics-Receives-FDA-Approval-of-Solosec-secnidazole-Oral-Granules-Following-Four-Year-Collaboration-with-Catalent).

Our understanding of the etiology, microbiology, therapeutic response, and recurrence following treatment and phenotypic outcome associated with BV is hampered by difficulty in diagnosis. As a result, self-diagnosis and self-treatment by symptomatic women are regularly employed and encouraged. In the UK, if a woman has a vaginal discharge that is abnormal for her, she generally assumes this is vulvovaginal candidiasis (VVC) until proven otherwise, despite the fact that BV is the commonest cause of vaginal discharge in high-income countries, responsible for one-third of all vaginal infectious morbidity and twice as common as VVC. In a study in three general practices in the UK, 287 asymptomatic women who attended for routine cervical cytology testing volunteered to provide an extra vaginal swab for VVC and BV analysis. Despite the fact that these women were asymptomatic and had attended their general practitioner’s (GP’s) surgery for routine cervical cytology rather than because they were symptomatic, 9% had BV and 3.8% had VVC. While this was not surprising, a significant percentage (7.7%) of those with BV had taken antifungals in the previous month (*P* = 0.028), indicating wrong self-diagnosis and self-treatment^12^.

### New information from molecular techniques

New information from molecular methods has added to our knowledge of vaginal eubiosis and dysbiosis^7^. By identifying organisms such as *L. iners* and *A. vaginae* that were previously under-detected and hence under-appreciated using cultivation techniques, we now know that there are different subtypes of vaginal eubiosis and dysbiosis, which may influence response to therapy and phenotypic outcome and cannot be discerned using vaginal Gram stain microscopy, the current gold standard for the diagnosis of BV. Accordingly, we need to develop and introduce novel diagnostics using molecular methods to address these shortcomings of current diagnostic techniques.

## Historical aspects of the diagnosis of bacterial vaginosis

### Non-molecular-based techniques

BV can be diagnosed clinically (a white/grey adherent vaginal discharge that is malodorous postmenstrually and postcoitally), by using Amsel composite clinical criteria^13^, microscopically by Gram stain of vaginal secretions to determine the Nugent score^14–20^, enzymatically^21–24^, chromatographically^25,26^, or using qualitative or semi-quantitative culture methods^27^. The pros and cons of the more commonly used techniques are shown in Table 1. The number of methods testifies to the fact that no single test is ideal, and that they can all provide false-positive and false-negative results. Several commercial point-of-care tests (POCTs) have been developed and introduced for the diagnosis of BV, though none are extensively used (Table 2).

**Table 1.** Commonly used non-molecular tests for the diagnosis of BV.

Diagnostic Test	Technique	Pros	Cons
Amsel criteria^13^	Identification of • characteristic white-grey, homogeneous, adherent vaginal discharge • pH >4.5 • positive “whiff test”* • Wet-mount microscopy to detect clue cells (≥20% per high-power field)**	A useful point-of-care test clinically	• Irreproducible • Subjective in the assessment of the four composite clinical criteria
Culture	Qualitative or semi-quantitative methods	Detection of *Gardnerella* *vaginalis* and other organisms	Lacks sensitivity and specificity, so the detection of *G. vaginalis* on culture is common in women with BV, but can also be present in a significant percentage of women who do not have BV^13,28^
**Vaginal** **Gram stain** **microscopy**			
Nugent scoring^14^	Quantitative assessment of *Lactobacilli,* *G. vaginalis*, and *Mobiluncus* spp. morphotypes using Gram stain microscopy (x1,000 magnification under oil-immersion) A score of • 0–3 = normal • 4–6 = intermediate • 7–10 = BV	Currently, the gold standard for the diagnosis of BV, more objective than Amsel	Needs trained and experienced microscopists
Ison/Hay method^19^	Same as Nugent but no quantitative measures • *Normal:* lots of *Lactobacilli* and few other morphotypes • *Abnormal:* lots of other morphotypes and few *Lactobacilli* • *Intermediate:* somewhere between the two	Simplifies Nugent scoring and is increasingly used in clinical practice and research. Doesn't require the same degree of training as Nugent	• Less objective than Nugent • Still requires some degree of microscopy training and experience

* Fishy, amine odor on application of the alkali potassium hydroxide ** Vaginal epithelial cells covered with coccobacilli that obliterate the cell margins and nuclei BV, bacterial vaginosis; POCT, point-of-care test

**Table 2.** Commercial point-of-care tests for the diagnosis of BV.

Commercial point-of-care test	Description	Sensitivity/specificity	Comments
Saling glove^29^	Fragment of narrow-range pH paper on the end of a finger of the disposable glove		Useful in population-based screening programs. If the pH is <4.5, then it is very unlikely the woman has BV. If the vaginal pH is ≥4.5, this could be due to the presence of blood (pH~7.4) or semen (pH~8.0) but would trigger further diagnostic tests for BV or other forms of vaginal dysbiosis.
Electronic nose^30^	Detection of volatile organic acids associated with BV by passing vaginal fluid over an array of specific sensors	Compared to Gram stain microscopy: 83%/77%	Similar tests based on pH and trimethylamine or proline amino- peptidase with mixed comparability to established tests exist but have failed to be introduced into regular usage^31^
BV Blue Test, Gryphus Diagnostics, AL, USA^22,32^	Chromogenic diagnostic test based upon detection of elevated sialidase (an enzyme produced by many anaerobes)	Good sensitivity, specificity, PPV, and NPV when compared to Amsel criteria and Nugent	Score was used clinically but failed to become a widespread diagnostic test for BV^22,32^
Affirm™ VP III, BD Diagnostics, Sparks, MD, USA^31^	Quantitative DNA probe for high concentrations of *Gardnerella* *vaginalis* in vaginal fluid for detection of *Candida* spp. and *Trichomonas vaginalis* in the same specimen	Sensitivity of 90% and specificity of 97% when compared with the detection of clue cells. Sensitivity of 94% and specificity of 81% when compared to a Nugent score of 7–10 on Gram stain microscopy. However, since *G. vaginalis* can be detected in up to 55% of women with no clinical signs of BV^13,28^, the test may be most useful in symptomatic patients or when used in conjunction with vaginal pH and a positive amine test. With these additional clinical criteria, the sensitivity increases to 97% and specificity to 71%.	Though it can be performed in an office setting, efficiency is better when carried out in the laboratory. With a 30–45 minute turnaround, promising results were obtained in pregnant and non-pregnant women as a supplement to Amsel criteria and Nugent scoring^31^.

BV, bacterial vaginosis; NPV, negative predictive value; PPV, positive predictive value

Existing diagnostic modalities are not well served by cultivation-dependent techniques and rely upon quantitative vaginal Gram stain microscopy that suffers from a degree of subjectivity. In the UK and continental Europe, in many laboratories, the microscopy is often performed by junior microbiology laboratory technicians who, following training and experience, are then promoted to more senior posts, and the responsibility for vaginal Gram stain diagnosis passes to new trainees. This is also probably why the PREMEVA Study provided negative results due to flaws in the diagnosis of BV. In their protocol, the investigators recognized that Nugent scoring was rare in France and medical laboratories would require training in the technique. To standardize the diagnosis of BV using Nugent scoring, technicians in 149 laboratories in 40 centers across France were given a training video, a technical brochure, and “training slides”, but no further details were provided. There was a significant delay of 5 years from presentation in abstract form in 2013 at the SMFM meeting to publication in 2018. In 2015, one of the PREMEVA co-authors admitted that the reason for the delay was because of concerns about the diagnosis of BV by Gram stain (personal communication to R.F.L. and J.S.J.). Their intention was to repeat the diagnosis using molecular diagnostic methods (personal communication to R.F.L. and J.S.J.), but in the published paper, this was not the case. Accordingly, this emphasizes that there is a need for a more robust and objective diagnostic test for vaginal eubiosis and dysbiosis.

### Molecular-based techniques

The background to the development and introduction of molecular-based diagnostic tests for the diagnosis of BV has been reviewed excellently elsewhere^31^. Together with a number of subsequent studies that have been supportive of the developments over traditional methods of diagnosing BV^33–36^, advances have been made, albeit they may be complementary to, rather than replace, culture-based techniques^37^. These can be summarized as follows. Using species-specific primers in 27 women with BV, and 46 without BV, a total of eight different bacteria were significantly associated with BV (*P* <0.001). These were BVAB-1 (now proposed to be renamed *Candidatus Lachnocurva vaginae*)^5^, BVAB-2, BVAB-3 (now renamed *M. indolicus*)^6^, *Gardnerella, Leptotrichia, Megasphaera*, and *Atopobium* species, and *Eggerthella-*like uncultured bacterium. With a few exceptions, the combination of bacterium-specific polymerase chain reaction (PCR) assays did not substantially improve sensitivity or specificity^38^. In a follow up study that addressed the diagnostic accuracy of these target organisms tested against Amsel criteria and Nugent score, PCR detection of one or more of these fastidious organisms was a more reliable indicator of BV than the presence of *G. vaginalis* alone (Table 3)^39^. Using Nugent score to classify 231 vaginal samples, quantitative real-time PCR using specific primers identified *G. vaginalis*, *Lactobacillus* species, *Mobiluncus curtisii*, *A. vaginae,* and *M. hominis*. Twenty samples were classified as BV, 44 as intermediate, and 167 as normal microbiota. Qualitative PCR comparisons between women with and without BV for these organisms are demonstrated in Table 4. Median concentrations of *A. vaginae, G. vaginalis, M. curtisii,* and *M. hominis* were significantly higher, and median concentration of *Lactobacilli* species was significantly lower in women with BV compared to those without^40^. To optimize the molecular methods for routine practice, an adjusted quantification was made by creating a threshold DNA level for the five organisms listed, and their predictive values are illustrated in Table 4. When the area under the curve (AUC) from the receiver operating characteristic (ROC) curves for bacterial molecular counts were applied prospectively for validation in 56 pregnant women, the negative predictive value (NPV) was 96% and the positive predictive value (PPV) was 99%^40^.

**Table 3.** Sensitivities and specificities of individual or combined organisms for the detection of bacterial vaginosis using molecular techniques^38,39^.

Organism	Sensitivity	Specificity	Comments
BVAB-1*	-	97.8%	
BVAB-2	-	95.7%	
BVAB-3**	-	97.8%	
*Gardnerella vaginalis*	100%	41%	
*Leptotrichia*	-	95.7%	
BVAB-1 & BVAB-3	-	100%	
BVAB-1 & *Megasphaera*	100%	-	
*Megasphaera* & one BVAB spp.	99%	89%	Tested against Amsel criteria
*Megasphaera* & one BVAB spp.	95.9%	95.7%	Tested against Nugent score

BVAB, bacterial vaginosis-associated bacterium * BVAB-1 now proposed to be renamed *Candidatus Lachnocurva vaginae*^5^ ** BVAB-3 has been re-named *Mageeibacillus indolicus*^6^

**Table 4.** Qualitative and quantitative polymerase chain reaction analysis of organisms for the prediction of BV^40^.

Organism	BV positive (%) (n = 20)	BV negative (%) (n = 167)	Sensitivity	Specificity	PPV	NPV	*P*-value
**Qualitative**							
*Gardnerella vaginalis*	19 (95%)	116 (69%)					0.001
*Atopobium vaginae*	19 (95%)	79 (47%)					0.001
*Mobiluncus curtisii*	13 (65%)	21 (13%)					0.001
*Mycoplasma hominis*	10 (50%)	22 (13%)					0.001
*Lactobacilli*	12 (60%)	141 (84%)					0.007
^*^Quantitative							
*G. vaginalis* >10^9^			90%	99%	14%		
*A. vaginae* >10^8^			50%	100%	19%		
*M. curtisii* >10^5^			45%	100%	38%		
*M. hominis* >10^6^			30%	98%	38%		
*Lactobacilli* >10^8^			44%	100%	8%		

*Adjusted for quantitation BV, bacterial vaginosis; NPV, negative predictive value; PPV, positive predictive value

### The combination of *A. vaginae* and *G. vaginalis* for the diagnosis of bacterial vaginosis

*A. vaginae,* in some habitats where sugars are a scarce source of energy, produces significant quantities of ammonia through peptidyl-peptidase activity. *Prevotella bivia* (formerly *Bacteroides bivia*) also produces ammonia, and this acts as a substrate to promote the growth of *G. vaginalis*^41–43^. Accordingly, since *A. vaginae* and *G. vaginalis* are frequently detected in association with BV, a number of authors examined the possibility of combining these two organisms as a means of diagnosing BV^38,40,44–46^. Using species-specific PCR for *G*. *vaginalis* and *A. vaginae* after obtaining 145 vaginal samples from healthy pregnant and non-pregnant women, researchers found that the comparative ROC analysis was poor, with an accuracy of 63–68%. In contrast, detection of the simultaneous presence of *A. vaginae* and *G*. *vaginalis* to diagnose BV had an accuracy of 87.8% (*P* <0.001), a sensitivity of 78%, and a specificity of 98%^46^. In another study, *A. vaginae* and *G. vaginalis* were highly sensitive for BV (sensitivities 96% and 99%, respectively), but *A. vaginae* was more specific than *G. vaginalis* (specificities 77% and 35%, respectively). *A. vaginae* was rarely detected without *G. vaginalis*, and in women in whom both organisms were detected, there were higher rates of recurrence (83%) than in women infected with *G. vaginalis* alone^47^. Ninety-six clinical samples sent for BV diagnosis were tested for *A. vaginae*, *G. vaginalis*, *M. mulieris*, and *Bacteroides fragilis* using real-time PCR. Twenty-eight samples (29%) contained *A. vaginae*, 26 of which (93%) also contained *G. vaginalis*. In the 68 samples without *A. vaginae*, only 7 (10%) contained *G. vaginalis*^45^. Using a quantitative threshold, 19/20 BV samples had a DNA level for either *A. vaginae* of ≥10^8^ copies/mL or *G. vaginalis* of ≥10^9^ copies/mL and 9/20 had both. The combination of an *A. vaginae* DNA level of ≥10^8^ copies/mL and a *G. vaginalis* DNA level of ≥10^9^ copies/mL demonstrated the best predictive criteria for the diagnosis of BV with excellent sensitivity (95%), specificity (99%), NPV (99%), and PPV (95%)^40^.

## Current commercially available molecular tests for the diagnosis of bacterial vaginosis in the USA and the European Union

New technologies employing molecular markers for BV have been developed to overcome the problems associated with the Amsel criteria, vaginal Gram stain microscopy, and POCTs. Molecular tests have the advantage of i) objectivity, ii) quantification, iii) detection of fastidious organisms, and iv) validity for self-obtained vaginal swabs. The techniques are dependent upon the detection of specific bacterial nucleic acids and are primarily available as direct probe assays, as already mentioned^31^, or nucleic acid amplification test (NAAT) assays. The commonest target for the identification of bacteria using molecular-based techniques is the small ribosomal subunit of the 16S rRNA gene, which is useful because it is present in all bacteria and has regions of conserved sequence that can be targeted by universal (broad-range), or specific primers. It also has areas of heterogeneity that can be used to identify bacteria or to infer phylogenetic relationships through comparisons with known organisms in sequence databases^7^. The 16S rRNA gene has a length of approximately 1,540 nucleotides and contains nine hypervariable regions: V1 to V9. These hypervariable regions exhibit variable degrees of sequence diversity among different bacterial genera. The V1–V2, V3–V4, or V4 regions are most often targeted in microbiota studies. Universal PCR primer sets are designed to amplify as many different 16S rRNA gene sequences from a wide range of bacterial species as possible. However, there are no suitable 100% conserved regions of the 16S rRNA gene available for PCR amplification^48^.

### Commercially available molecular diagnostic tests for the diagnosis of bacterial vaginosis currently used in the USA

Currently in the USA, there are a number of tests for the molecular diagnosis of BV in symptomatic women, most if not all of which are multiplex PCR assays. While some of these laboratories have a small throughput, to our knowledge, there are five commercially available molecular diagnostic tests for the diagnosis of BV that dominate the market in the USA^49,50^, two of which are FDA cleared^50,51^: i) NuSwab (Laboratory Corporation of America Holdings, Burlington, NC, USA)^49^, ii) SureSwab (Quest Diagnostics, Secausus, NJ, USA), iii) BD MAX™ Vaginal Panel (VP) (Becton Dickinson, Sparks, MD, USA)^51^, iv) MDL BV Panel (Medical Diagnostics Laboratory, Hamilton Township, NJ, USA), and v) Hologic Aptima® BV Assay (Hologic Inc., San Diego, CA, USA)^50^. These are NAATs that are capable of detecting as little as one organism per sample. In a short period of time, specific nucleic acid sequences are enzymatically amplified exponentially to produce billions of copies of the sequences. The amplified products are detected and identified. Such real-time PCRs eliminate the need for post-amplification analysis and reduce the risks of contamination. The bacteria that are positive or negative predictors of BV and other microorganisms detected in the various tests and the test characteristics and comparator standards are listed in Table 5 and Table 6, respectively.

**Table 5.** The bacteria and other microorganisms detected and used to diagnose BV and other causes of vaginal dysbiosis.

	NuSwab^**^	SureSwab	BD MAX Vaginal Panel^***^	MDL BV Panel	AmpliSens^*^	Hologic Aptima BV
**POSITIVE PREDICTORS OF BV**						
*Atopobium vaginae*	✓	✓	✓	✓	✓	✓
*Gardnerella vaginalis*		✓	✓	✓	✓^*^	✓
*Megasphaera spp.* (types 1 or 2)	✓	✓	✓	✓		
BVAB2	✓		✓			
**NEGATIVE PREDICTORS OF BV**						
*Lactobacillus acidophilus*		✓			^*^	
*Lactobacillus crispatus*	✓	✓	✓		^*^	✓
*Lactobacillus jensenii*		✓	✓		^*^	✓
*Lactobacillus gasseri*						✓

BV, bacterial vaginosis; BVAB, bacterial vaginosis-associated bacterium * The AmpliSens assay was set up to detect the genus *Lactobacillus* rather than specific species of *Lactobacilli*. *G. vaginalis* clades 1 and 2 and A. vaginae were detected, and total bacteria for the diagnosis of BV was used. ** An extended version also detects i) *Candida albicans*, ii) *Candida glabrata*, iii) *Chlamydia trachomatis*, iv) *Neisseria gonorrhea*, and v) *Trichomonas vaginalis.* *** Also detects i) *T. vaginalis*, ii) Candida Group (*C. albicans, Candida tropicalis, Candida parapsilosis*, and *Candida dubliniensis*), iii) *C. glabrata*, and iv) *Candida krusei*

**Table 6.** Characteristics and comparator standards of FDA-approved^§^ and CE-IVD marked^¶^, commercially available, molecular diagnostic tests (using quantitative multiplex PCR assays) for the diagnosis of BV^⌘^.

	Sensitivity	Specificity	PPV	NPV	Comparator standard
**NuSwab**^52^	96.7%	92.2%	94%	95.6%	Combination of Amsel and Nugent criteria
**SureSwab**	_	_	_	_	Results interpreted according to data from Quest Diagnostics^49^
^¶^^§^ **BD MAX Vaginal** Panel^**^^51^	90.5–96.2%	85.8–96.5%	89%	87.7%	Variably, a number of standards including Amsel, Nugent, and Ison/Hay criteria, culture, microbiota analysis, and the Affirm test
**MDL BV Panel**^53^	99%	94%	94%	94%	Combination of Amsel and Nugent criteria
^¶^ AmpliSens^*^^54^	97.5%	76.8%	77.5%	97.3%	Clinical diagnosis of BV using symptoms, vaginal pH, and wet mount/Gram stain microscopy
^¶^^§^ Hologic Aptima BV^***^^50^	95–97.3%	85.8–92.4%	_	_	Nugent criteria (plus Amsel if intermediate Nugent score)

BD, Becton Dickinson; BV, bacterial vaginosis; CE-IVD, Conformité Européene *in vitro* diagnostics; FDA, Food and Drug Administration; MDL, Medical Diagnostics Laboratory; NPV, negative predictive value; PCR, polymerase chain reaction; PPV, positive predictive value * Characteristics of the test are based on a subgroup of symptomatic women ** The range of sensitivities and specificities is derived from a number of different studies that used different standards as comparators *** Clinician-collected and patient-collected specimens had similar performance § FDA approved ¶ CE-IVD marked ⌘ This table presents separate data from unrelated studies, not comparative differences from a single study or a systematic review or meta-analysis, and does not distinguish between the quality or strength of evidence

The NuSwab detects *A. vaginae*, BVAB-2, and *Megasphaera* spp., which are predictive of BV, and *L. crispatus*, which is a negative predictor of BV^7^. *G. vaginalis* was not included because *G. vaginalis* can be detected in up to 55% of women with no clinical signs of BV^13,28^. An extended version detects i) *Candida albicans;* ii) *Candida glabrata;* iii) *C. trachomatis*, iv) *N. gonorrhea*. and v) *Trichomonas vaginalis* (TV)^52^ (see https://files.labcorp.com/testmenu/180021.pdf).

The SureSwab detects i) *G. vaginalis*, ii) *A. vaginae*, and iii) *Megasphaera* spp. as positive predictors of BV and iv) *L. crispatus*, v) *L. acidophilus*, and vi) *L. jensenii* as negative predictors of BV. Sensitivity, specificity, PPV, and NVP were not reported, as the complexity of a BV diagnosis is based upon certain indicator organisms and is an example of how the reported result is interpreted according to data from Quest Diagnostics (Secaucus, NJ, USA)^49^ (see https://testdirectory.questdiagnostics.com/test/test-detail/17333/sureswab-vaginosisvaginitis-plus?cc=MASTER).

The BD MAX™ VP is the first FDA market-authorized, microbiome-based PCR assay that directly detects the three most common infectious causes of vaginitis: i) BV, ii) VVC, and iii) trichomoniasis (also known as TV). For the diagnosis of BV, *G. vaginalis, A. vaginae, Megasphaera* type-1, and BVAB-2 are used as positive predictors of BV and *L. crispatus and L. jensenii* are used as negative predictors of BV. The proprietary algorithm provides a laboratory report as positive or negative for BV. The test also detects i) TV, ii) Candida group (*C. albicans, Candida tropicalis, Candida parapsilosis*, and *Candida dubliniensis*), iii) *C. glabrata*, and iv) *Candida krusei*^51^.

The future also holds hope for new technologies such as machine-learning/artificial intelligence. In a recent study, a machine-learning-based algorithm of seven bacterial strains was measured against the Ison/Hay criteria^19^ and was found to have excellent specificity (98%) and NPV (95%)^55^.

The MDL BV Panel targeted nine vaginal bacteria as potential markers for BV. Using quantitation and ROC curves, three organisms were chosen as predictors of BV: i) *G. vaginalis*, ii) *A. vaginae*, and iii) *Megasphaera* types 1 and 2. *L. crispatus* was left out of the final model because it did not contribute to diagnostic accuracy^53^ (see https://www.mdlab.com/forms/TechBulletin/BV_Panel_Lactobacillus.pdf).

The Hologic Aptima® BV assay is an *in vitro* NAAT that uses real-time transcription-mediated amplification for the detection and quantitation of ribosomal RNA from bacteria associated with BV. Two organisms were chosen as positive predictors of BV (*G. vaginalis* and *A. vaginae*) and three species of *Lactobacilli* (*L. gasseri, L. crispatus,* and *L. jensenii*) were chosen as negative predictors of BV. The assay reports a qualitative result for BV rather than results for individual organisms^50^ and is intended to aid in the diagnosis of BV on the automated Panther® system using clinician- and patient-collected vaginal swabs from women with signs and symptoms of vaginitis and/or vaginosis (see https://www.hologic.com/hologic-products/diagnostic-solutions/aptima-vaginal-health).

### Tests approved for use in Europe

To our knowledge, currently in Europe, there are three commercially available molecular diagnostic tests for the diagnosis of BV, all of which are multiplex PCR assays that are CE-IVD marked, are fast, and have a high sensitivity and specificity^50,51,54^. Two of these (BD MAX™ VP and Hologic Aptima® BV assays) have already been outlined above. The other is the AmpliSens® (sometimes referred to as the ATRiDA test) Florocenosis/Bacterial vaginosis-FRT PCR test (InterLabService, Moscow, Russia). The AmpliSens® test measures the relative concentration of i) *Lactobacillus* spp., ii) *G. vaginalis* clades 1 and 2, iii) *A. vaginae*, and iv) total bacteria to diagnose BV.

### Comparative testing

The accuracy of a commercially available multiplex PCR (ATRiDA) for the diagnosis of BV was evaluated in women reporting urogenital symptoms and women notified for STIs who were not necessarily symptomatic. The ATRiDA test (ATRiDA B.V., Amersfoort, the Netherlands) targets *G. vaginalis*, *A. vaginae*, and *Lactobacillus* spp. in relation to overall bacterial load. The ATRiDA test outcome was compared to the clinical BV diagnosis and to vaginal microbiota composition, determined by 16S rRNA gene sequencing, which enables accurate characterization of complex microbial communities with respect to membership and their relative abundance to each other. Based on statistical analyses of vaginal microbiota data, BV has been defined as ≤47% relative abundance of *Lactobacillus* spp. and an increased presence of anaerobes. Although recommended by some, microbiota analysis is currently quite laborious and expensive for it to be used routinely in clinical practice^56,57^.

Overall, compared to the clinical BV diagnosis, the ATRiDA test demonstrated high sensitivity (96.9 %) and moderate specificity (70.2 %). The NPV was >97.3% and the PPV differed by study group but was highest in women reporting urogenital symptoms (78.2 %). 16S rRNA gene sequencing showed that 54 % of women who were ATRiDA BV positive but clinically asymptomatic had a diverse anaerobic vaginal microbiota, classified as asymptomatic vaginal dysbiosis. The authors concluded that the ATRiDA test was a sensitive method for the detection of BV but advised that a positive test should be interpreted in conjunction with clinical symptoms^58^.

Recently, two CE-IVD-marked real-time PCR assays (AmpliSens® and BD MAX™ VP) were compared with Amsel criteria, Nugent score, and/or culture for the diagnosis of BV. Microbiota analysis, based on amplicon sequencing of the 16S rRNA gene, described elsewhere^59^, was used as a reference test. Based on the microbiota analysis, the sensitivity of detecting BV was 38.9% for culture, 61.15% for Amsel criteria, 63.9% for Nugent score and the BD MAX™ VP assay, and 80.6% for the AmpliSens® assay. The specificity of all methods was ≥92.4%. The authors concluded that, compared to Amsel criteria, Nugent score, culture, and the BD MAX™ VP assay, the AmpliSens® assay offered optimal agreement with microbiota analysis. However, concerns were expressed that the handling of samples was not performed in accordance with the manufacturer’s instructions for the BD MAX™ VP assay. The eSwabs were tested with the BD MAX™ VP assay by pipetting 500 μl of eSwab into 500 μl of BD MAX™ Sample Tube Buffer. Although this was done in consultation with BD, it might have influenced the sensitivity of the assay. Furthermore, the report did not describe the advantage of the BD MAX™ VP assay to test for BV, VVC, and TV as a single test. For the diagnosis of VVC, the performance of the BD MAX™ VP assay was comparable to other applied methods and detected additional positive samples compared to the current gold standard (fungal culture). None of the included samples was positive for TV by the applied assays, including the BD MAX™ VP assay. One of the classifications was for aerobic vaginitis (AV) as a separate identity from BV, the significance of which, particularly as a predictor of PTB, has been questioned^60^.

Another recent study evaluated the BD MAX™ VP for the diagnosis of BV, VVC, and TV. The test was compared with i) a combination of Ison/Hay criteria^19^, the presence of clue cells, and a significant growth of *G. vaginalis*, ii) yeast culture, and iii) a combination of culture, wet mount microscopy, and an alternative molecular assay^61^. The sensitivity and specificity for BV was 89.8% and 96.5%, respectively. For TV, the sensitivity and specificity were both 100%, and for VVC, the sensitivity and specificity were 97.4% and 96.8%, respectively. The authors concluded that the BD MAX™ VP was highly sensitive and specific and simplified the identification of vaginitis of infectious origin.

Finally, in a group of 200 symptomatic women, the performance of the BD MAX™ VP was compared to that of the Affirm test, which, for the purpose of the study, was considered to be the standard of care. Both multiplex assays are commercially available for the detection of DNA from organisms associated with vaginitis, including BV, VVC, and TV. The sensitivity and specificity of the BD MAX™ VP for BV was 96.2% and 96.1%, respectively, compared to 96.2% and 81.6%, respectively, for the Affirm test. The sensitivity and specificity of the BD MAX™ VP for VVC was 98.4% and 95.4%, respectively, compared to 69.4% and 100%, respectively, for the Affirm test. The BD MAX™ VP and Affirm test demonstrated 100% concordance for the detection of TV. The authors concluded that the results demonstrated an improvement in accuracy of the BD MAX™ VP compared to the Affirm test for the detection of BV and VVC^62^. Using Nugent scoring as a reference, three molecular assays were assessed for the diagnosis of BV, examining the impact of incremental increases in bacterial targeting. The introduction of *A. vaginae* improved specificity for the diagnosis of BV, but co-infection with VVC was common (13.5%)^63^, and the shortcomings of molecular-based techniques in the presence of mixed vaginal infections have been emphasized^64^.

In a recent Swedish study of women undergoing termination of pregnancy, BV was determined to be present or absent on the basis of a modified Ison/Hay criteria assessment^19^ and compared with a molecular test analyzing six different bacteria associated with BV (*A. vaginae*, BVAB-2, *G. vaginalis*, *Leptotrichia/Sneathia* spp., *Megasphaera* spp., and *Mobiluncus* spp.) in relation to *Lactobacillus* spp. using real-time PCR. There was excellent agreement between the compared methods, with a kappa coefficient value of 0.87 (0.76–0.99). Compared to the modified Ison/Hay criteria, the molecular test achieved a sensitivity of 91%, specificity of 97%, PPV of 91%, and NPV of 97%^65^.

### Emerging techniques and tests

Microarray analysis and next-generation sequencing (NGS) have contributed to our understanding and development of commercially available tests for BV, but, as yet, these assays are generally performed in large reference laboratories and, outside North America, such as the UK and continental Europe, are not yet commercially available as a routine option^49^.

### Use of molecular diagnostic tests in a clinical setting

**Figure 1.**
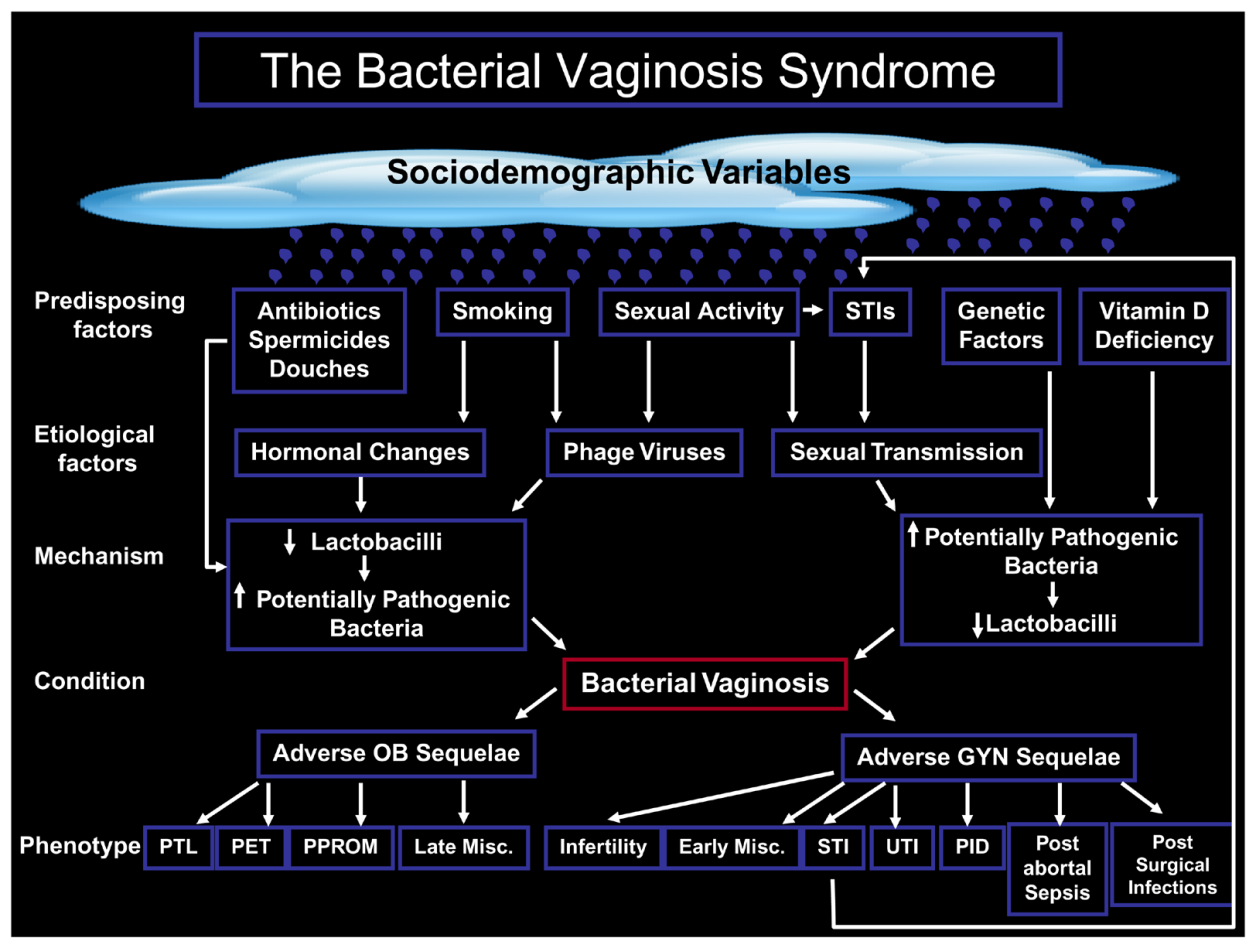
The proposed interaction among the mechanisms, etiology, predisposing factors, and phenotypic outcomes in obstetrics and gynecology of “the bacterial vaginosis syndrome”. GYN, gynecological; Misc, miscarriage; OB, obstetric; PET, pre-eclampsia; PID, pelvic inflammatory disease; PPROM, preterm prelabor rupture of the membranes; PTL, preterm labor; STI, sexually transmitted infection; UTI, urinary tract infection.

Women with symptoms of vaginal dysbiosis may present to their GP, a genitourinary medicine (GUM) clinic, a family planning clinic, or directly to a gynecologist, among whom the services offered and the management and diagnostic skills such as microscopy may differ^66^. The etiology of “the BV syndrome” remains unknown. BV is the most common cause of vaginal discharge globally, with an estimated annual economic burden of $4.8 billion^7,67,68^. BV has important public health implications such as PTB and increased risk of acquisition of STIs such as gonorrhea, chlamydia, TV, HSV, and HIV^69^. These are associated with serious mortality and morbidity for women and babies. The place of the condition as a syndrome, together with the proposed potential mechanisms, suggested etiological and predisposing factors, overlying sociodemographic variables, and the different phenotypic outcomes are presented in Figure 1. Though the BV syndrome is certainly sexually associated, being increased in women with i) early sexual debut, ii) greater number of lifetime sexual partners, and iii) introduction of a new sexual partner, increasingly the epidemiology of some subtypes of the BV syndrome supports sexual transmission^70^. At the center of the debate is whether or not BV is caused by a primary pathogen or a polymicrobial, synergistic consortium of microorganisms that are sexually transmitted^71^.

A conceptual model^72^, which was recently updated, suggested that BV originates through the sexual transmission of *G. vaginalis*^73^. Using its virulence factors, it successfully colonizes the host’s vaginal epithelium and forms a biofilm community to overcome *Lactobacilli*. *G. vaginalis*’s genetic diversity may lead to both pathogenic and non-pathogenic strains. Symbiotic relationships with normally dormant vaginal anaerobes lead to increases in the latter, which contribute to the symptoms of BV. Critics of this model point out that *G. vaginalis* can be found in a *virgo intacta* and in sexually active women with a healthy vaginal microbiota and that colonization with *G. vaginalis* does not always lead to BV^74^, though this has been challenged^75^. Nevertheless, recent research into the pathogenesis of BV has determined the existence of a number of different species within the *Gardnerella* genus. It may be that healthy women are colonized by non-pathogenic species of *Gardnerella*, whereas virulent strains are involved in the development of BV^72^.

To address this problem, the likelihood of STIs was assessed in symptomatic women tested for vaginal dysbiosis using both the BD MAX™ VP and the BD MAX™ CT/GC/TV molecular diagnostic assays. Positivity rates for *C. trachomatis, N. gonorrhoeae*, and TV DNA were calculated, and concordance rates between the assays for the detection of TV were determined. Women with BV alone or with concurrent VVC had high rates of co-infection with STIs (24.4–25.7%). TV results were concordant between the two assays in 559 of 560 samples tested. These data suggest that women with symptoms of vaginal dysbiosis in the form of BV or candidiasis may be at risk of an STI. Accordingly, molecular testing may provide a broader diagnostic coverage for symptomatic women regardless of the type of clinic in which they are seen^76^.

Symptomatic women attending a GUM clinic in the UK had two vulvovaginal swabs taken. One was used for a chlamydia and gonorrhea NAAT and one for testing on the BD MAX™ VP. Speculum examination was then performed and vaginal swabs were taken for vaginal pH and examination by wet-mount and Gram stain microscopy for VVC, BV, and TV. Forty-six (23.6%) women were negative for all three infections on the BD MAX™ VP. Ninety-three were positive for BV (47.7%), 70 (35.9%) for VVC, and 9 (4.6%) for TV. Thirty-six women tested positive for both BV and VVC on the BD MAX™ VP. The test sensitivity for VVC was 86.4% and the specificity was 86.0%. For BV, the sensitivity was 94.4% and the specificity was 79%. While the sensitivity for BV was encouraging, the specificity was lower than expected. This may reflect the higher rate of STIs in the study population, which worsened the vaginal dysbiosis. The author concluded that while NAATs do not provide immediate results, they were easy to process and offered advantages over a high vaginal swab performed in other settings where rapid microscopic assessment was not readily available^77^. Since such tests are simple, convenient, reliable, and inexpensive, perhaps every woman suspected of having BV should have a simultaneous molecular test (PCR or gene sequencing) for other STIs, albeit this may depend on the patient’s risk factors.

## Discussion

Currently, there are a number of diagnostic tests available to diagnose BV, ranging from POCTs to molecular assays. The choice of test will be influenced by the cost, availability, speed, and accuracy of the test. There are few recommendations with respect to the use of molecular-based technology to screen for BV, especially in asymptomatic women^49^. A recently updated ACOG practice bulletin on vaginitis was released^78^ that applied to BV, VVC, and TV. This still recommends traditional methods for diagnosing vaginitis such as culture, Amsel criteria, and Nugent score. While the bulletin acknowledges that PCR diagnostic tests exist and can be considered for use, it states that they have “comparable sensitivity and specificity to Nugent scoring and Amsel criteria”, citing three studies^51,61,79^. However, to justify this statement, at least one of the studies cited actually showed an improvement in performance of NAAT compared to Amsel criteria and clinician diagnosis^79^. Furthermore, a recent study using a different NAAT to test for BV demonstrated a similar improvement compared to Amsel criteria and clinician diagnosis^45^. Many researchers agree that molecular assays are superior to conventional methods such as Amsel, Gram stain, and DNA probes like the Affirm test. What is difficult to explain is why they are not in more widespread use in clinical practice. This is almost certainly a problem in educating the general public, primary care physicians, and specialist clinicians of the potential benefits of these technologies. In the UK, our experience is of academic centers testing and validating the technology, following which they are keen to introduce the technology, but a business case has to be made, which takes time with budgets planned a year in advance. In addition, the COVID-19 pandemic has disrupted research, clinical care, and funding in other areas.

In the USA, it is our understanding that the acknowledgement of the benefits of NAATs suffers from the dynamics between regulatory guidelines and payer/reimbursement. While the ACOG guidelines acknowledge the role of NAATs, they do not provide a firm endorsement. Since the funders of healthcare rely heavily on such guidelines, their policies do not support full reimbursement of NAAT for BV. Simultaneous NAAT for STIs is likely to provide further resistance. It is likely to require stronger endorsement in the guidelines of the ACOG or the CDC for NAATs to diagnose BV, TV, VVC, and other STIs to be funded.

This review is designed to increase knowledge about the different molecular assays available, the range of targeted microorganisms, and the test characteristics with respect to sensitivity, specificity, PPV, and NPV. Nevertheless, since BV is a polymicrobial condition, diagnosed (whether by molecular or microscopy techniques) by the presence of potentially dysbiotic organisms and the absence of eubiotic organisms, investigators should avoid the use of direct probe assays of single BV indicator organisms such as *G. vaginalis* in favor of multiplex PCR technology that is able to detect multiple indicator (or contra-indicatory) organisms.

### Nugent’s intermediate category

None of the molecular-based tests discussed address the “intermediate” category on Nugent’s score^14^ (grade II; score 4–6), unless they consider that reports such as “unspecified dysbiosis” or “equivocal” or “unclassified” are synonymous for Nugent’s intermediate category. It is important to recognize that this intermediate category is a Gram-stain diagnosis, rather than one that is made clinically, and that this category is a distinct entity not only because amalgamation with grade III (BV; Nugent score 7–10) diminishes the specificity and PPV of the Gram-stain for diagnosing BV but also because women with grade II Nugent score often fail to respond to clindamycin, whereas those of grade III do not. Although a distinct entity, the intermediate category is likely to be a transient state on a continuum from a normal microbiota to BV and *vice versa*^80,81^. Our recommendation would be that if the result of the test returns as i) unspecified dysbiosis, ii) equivocal, iii) unclassified, iv) intermediate, or v) indeterminate, repeat the test after a short interval of 10–14 days, and if the result is still unclear and the patient is symptomatic, they should be treated.

### *L. iners*: relative abundance and diversity

There is increasing interest in the vaginal microbiome as a biomarker for PTB^9^, and *L. iners* may play an important part in the etiology of PTB^9,82,83^. None of the molecular diagnostic tests discussed above targeted *L. iners.* This is understandable, since the presence or absence of *L. iners* does not differentiate between vaginal eubiosis and dysbiosis. While *L. iners* is commonly found in BV as well as in the eubiotic vagina, it is rare to find one of the other “big three” (*L. crispatus, L. jensenii*, and *L. gasseri*) in abundant numbers in the presence of BV and, conversely, it is rare to find BV when these three species of *Lactobacilli* are found in abundance in the presence of minimal diversity of other potentially pathogenic organisms. Nevertheless, the targeting of *L. iners* might improve sensitivity and specificity if the relative abundance of *L. iners* and the diversity of other organisms were known and taken into consideration. In a study of the vaginal microbiome in women with PPROM^84^, using relative abundance and diversity, it was possible to differentiate between the presence of *L. iners* in a eubiotic as well as a dysbiotic vaginal microbiota. In a eubiotic vaginal microbiota, the diversity of organisms is as low as two or three organisms, whereas in BV, the diversity is significantly higher (eight or more)^7^. In a vaginal microbiota that was dominated by an abundance of *L. iners,* with or without *L. crispatus, L. gasseri,* or *L. jensenii*, where there was minimal diversity of other organisms in the microbiota, this could be classified as eubiotic. In contrast, in a vaginal microbiota that was dominated by an abundance of *L. iners,* in the absence of any other species of *Lactobacilli,* and a high level of diversity (eight or more other potentially pathogenic organisms), this was clearly dysbiotic (unpublished data).

### Clades, biovars, and quantitation

The propensity of *G. vaginalis*, compared to other BV-associated bacteria, to form a biofilm that provides a scaffold to which other potentially pathogenic bacteria can attach^85–88^ may be very important. While the presence or absence of *G. vaginalis* has importance, quantitative analysis is more important with both traditional cultivation-independent^89^ as well as novel molecular-based techniques^56^. These demonstrate that a heavy growth, rather than simply presence or absence, is more predictive of pathology that leads to adverse sequelae^56,89^ and emphasizes the importance of quantitative PCR analysis (see sections on molecular-based techniques and combining *A. vaginae* and *G. vaginalis* for the diagnosis of BV above).

Based on enzyme activity and biochemical usage of various sugars, *G. vaginalis* can be identified as a number of biotypes that render the species phenotypically and genetically heterogeneous. Phenotypic diversity within the species is manifest by i) virulence factors, ii) cytotoxicity, iii) production of lytic enzymes such as vaginolysin and sialidase that break down the local secretory IgA host response, iv) biofilm formation, v) susceptibility to antibiotics, and vi) adhesion and ability to dislodge species of *Lactobacilli* from vaginal epithelial cells^56^. Genomic analysis of species of *G. vaginalis* has identified four clades (1–4). A clade is defined as “a group of organisms believed to comprise all the evolutionary descendants of a common ancestor”. An example is the *Hominids*, a clade occupied by humans, chimpanzees, gorillas, orangutans, and gibbons. The four clades of *G. vaginalis* are different with respect to genome size and G:C (guanine:cytosine) ratio, sufficient for them to be considered as separate species. In a study that aimed to evaluate the distribution and abundance of the *G. vaginalis* clades and sialidase production, the quantification of all four *G. vaginalis* clades discriminated between a BV microbiota and a normal microbiota more accurately than measuring *G. vaginalis* sialidase production. Clade 4 was strongly associated with a BV microbiota, despite most clade 4 strains lacking the sialidase A gene. These genetic differences among strains might identify potentially pathogenic from commensal strains of *G. vaginalis*^56,90–93^ and the host’s response to each^94,95^. For further information, the reader is guided to an excellent review^4^. These factors may be important in the choice of clades of *G. vaginalis* targeted in molecular-based tests for BV and vaginal dysbiosis^54,58^.

The phenotypic and genotypic variation among strains of *G. vaginalis* may mirror the confusion over the role of the genital mycoplasmas in adverse outcomes of pregnancy^89,96^. Genital species of *Ureaplasma* are detected frequently in healthy, asymptomatic individuals. Using cultivation-dependent techniques in women delivering between 26 and 34 completed weeks of gestation, 80% and 24% of those in spontaneous labor were colonized by *Ureaplasma urealyticum* and *M. hominis*, respectively. In contrast, in women not in labor but delivered electively for fetomaternal indications at the same gestational age, 46% and 8%, respectively, were colonized by *U. urealyticum* and *M. hominis*^89,97^. This qualitative analysis was significantly different for *U. urealyticum* (*P* <0.01) but not for *M. hominis.* Nevertheless, when only a heavy colonization of *M. hominis* was considered (>10^5^ color change units [the unit of quantity at the time]), 13 women in the study group (18%) and none of the controls were identified (*P* <0.05), suggesting that it was not the presence or absence of the organism that was important but the quantity present^89^. Only certain subtypes of the species are pathogenic. Using molecular-based techniques, it was found that *Ureaplasma* species consist of 14 serovars from two biovars. The majority of human *Ureaplasma* isolates belong to *Ureaplasma parvum* (biovar 1) comprising four serovars (the predominant biovar in patients with genital tract infections), with *U. urealyticum* (biovar 2) comprising 10 serovars isolated much less often. However, the data are limited and conflicted because of the difficulties with traditional genotyping methods^98^. This may be relevant to the confusion about the role of *U. urealyticum* in adverse outcomes of pregnancy^96^.

The genital mycoplasmas have been implicated in a number of adverse outcomes in obstetrics and gynecology, and their role is complicated by the presence or absence of BV^96,99^. *Mycoplasma genitalium* is an STI that shares similar clinical aspects with *C. trachomatis* and should be treated if detected. Nevertheless, further research is necessary before *M. genitalium* is included in molecular-based tests for BV.

## Conclusions

New information from molecular-based cultivation-independent techniques has identified candidate organisms for inclusion in multiplex diagnostic tests. This review presents separate data from unrelated studies, not comparative differences from a single study or a systematic review or meta-analysis. Such diagnostic techniques demonstrate high sensitivities, specificities, PPVs, and NPVs against existing gold-standard diagnostic techniques and will hopefully continue to improve, as our understanding of the vaginal microbiome expands. However, further development and refinement are necessary, and care must be taken when measuring these parameters against different BV diagnostic standards in different molecular diagnostic tests. Testing needs to reflect the importance of the absence of negative markers of BV such as *L. crispatus* as much as the presence of positive markers such as the combination of *G. vaginalis* and *A. vaginae.* They should also take into consideration quantitative as well as qualitative analysis. While they cannot account for rarely detected organisms whose role in the BV syndrome remains unknown, they must continue to study these organisms and make clinicians aware of the false negatives and shortcomings of the test because these organisms may not have been incorporated. It is important to recognize and investigate further the role of *L. iners* in vaginal eubiosis and dysbiosis. Presence or absence is not as important as relative abundance and diversity among other organisms present when differentiating between eubiosis and dysbiosis in the case of *L. iners.* Future tests should recognize and incorporate this anomaly. The choice of primers used for studying the vaginal microbiota has important implications on the evaluation of NGS of the vaginal microbiota using partial 16S rRNA gene amplicons and has been discussed elsewhere^9^. Primers spanning the V3–V4 region identify more taxa in the vaginal microbiota than those from the V1–V2 region, particularly taxa such as *G. vaginalis.* This is also important when choosing which clade of *G. vaginalis* or biovar of *Ureaplasma* is being targeted. The future also holds hope for new technologies such as machine-learning and artificial intelligence.

## Abbreviations

BV, bacterial vaginosis; BVAB, bacterial vaginosis-related bacterium; CE-IVD, Conformité Européenne *in vitro* diagnostic; CST, community state type; FDA, Food and Drug Administration; GAIN, Generating Antibiotic Innovation Now; GP, general practitioner; GUM, genitourinary medicine; HIV, human immunodeficiency virus; HSV, herpes simplex virus; NAAT, nucleic acid amplification test; NGS, next-generation sequencing; NPV, negative predictive value; PCR, polymerase chain reaction; POCT, point of contact test; PPROM, preterm prelabor rupture of the membranes; PPV, positive predictive value; PTB, preterm birth; ROC, receiver operating characteristic; STI, sexually transmitted infection; TV, *Trichomonas vaginalis*; VP, vaginal panel; VVC, vulvovaginal candidiasis.
